# Clinical Features of Parkinson's Disease in Patients with Early-Onset Freezing of Gait

**DOI:** 10.1155/2022/4732020

**Published:** 2022-07-08

**Authors:** Hiroo Terashi, Yuki Ueta, Takeshi Taguchi, Hiroshi Mitoma, Hitoshi Aizawa

**Affiliations:** ^1^Department of Neurology, Tokyo Medical University, 6-7-1 Nishishinjuku, Shinjuku-ku, Tokyo 160-0023, Japan; ^2^Department of Medical Education, Tokyo Medical University, 6-7-1 Nishishinjuku, Shinjuku-ku, Tokyo 160-0023, Japan

## Abstract

**Background:**

Freezing of gait (FOG) is an important symptom that can impair activities of daily living in patients with Parkinson's disease (PD). However, its pathogenic mechanism is largely unknown. The aim of the present study was to elucidate the clinical characteristics of newly diagnosed and levodopa-naïve patients with PD who present with FOG.

**Methods:**

A total of 53 patients with untreated PD (29 men and 24 women) within 2 years of disease onset were included in the study. Using item 3 of the Freezing of Gait Questionnaire (FOG-Q), patients were classified as “freezers” and “nonfreezers” and compared for cognitive function, depressive symptoms, apathy, olfactory function, motor severity, gait parameters, and daily physical activity. We also assessed the relationship between FOG severity (total score of items 3–6 on the FOG-Q) and various clinical parameters.

**Results:**

The FOG was reported by 8 (15%) patients with PD. The Apathy Scale score (*p*=0.018), Modified Hoehn and Yahr stage (*p* < 0.001), Unified Parkinson's Disease Rating Scale part III score (*p* < 0.001), and postural instability and gait disorder score (*p* < 0.001) were significantly higher, and the mean gait acceleration amplitude (*p*=0.006) was significantly lower in freezers compared to that in nonfreezers. However, there was no significant correlation between FOG severity and these clinical parameters. There was also no significant difference in cognitive function, depressive symptoms, and olfactory function between the two groups. Daily physical activity was significantly lower in freezers than that in nonfreezers.

**Conclusions:**

Since FOG develops soon after PD onset, the study findings suggest that the FOG might be associated with the severity of apathy, motor symptoms, and in particular, gait disturbance.

## 1. Introduction

Freezing of gait (FOG) is a characteristic gait disturbance defined as a “brief, episodic absence, or marked reduction of forward progression of the feet despite the intention to walk” [1]. The prevalence of FOG in Parkinson's disease (PD) correlates with disease duration, reportedly being 6% in the first year, ∼40% within 10 years, and ∼80% within 20 years of disease onset [2, 3]. Furthermore, the relative risk of FOG in PD is higher in patients with disease onset at age ≥70 years [4]. FOG is an important symptom because it can impair activities of daily living (ADLs) and the health-related quality of life of patients with PD. However, its mechanism is largely unknown [5, 6]. Earlier studies reported a close correlation between the FOG onset and the left-sided disease onset, severity of motor symptoms, especially axial symptoms, and severity of nonmotor symptoms, such as cognitive impairment, mood disorders, sleep disturbance, and autonomic failure, although definitive consensus has not been attained [1, 7, 8]. While many studies have investigated the FOG in PD, few focused on FOG in an early-stage PD. As it is currently unclear whether FOG pathology differs depending on the stage of PD, knowing the association between FOG and the PD stage is important in understanding the mechanism of FOG.

The aim of the current study was to elucidate the clinical characteristics of newly diagnosed and levodopa-naïve patients with PD presenting with FOG.

## 2. Materials and Methods

### 2.1. Participants

This study included patients with idiopathic PD who visited the Outpatient Clinic of the Department of Neurology, Tokyo Medical University Hospital, between January 2014 and October 2019 and met the following inclusion and exclusion criteria. The inclusion criteria were as follows: (1) age <80 years, (2) within 2 years of the appearance of motor symptoms, (3) no prior treatment with antiparkinsonian drugs, (4) Mini-Mental Status Examination (MMSE) score ≥20, (5) availability of data on gait analysis recorded using a wearable sensor, and (6) provided a signed consent form for participation in the study. The exclusion criteria were as follows: (1) concomitant neurodegenerative disease, (2) history of treatment for psychiatric disorders, (3) history of stroke, and (4) presence of joint pain or spinal disease interfering with activities of daily living. PD diagnosis was based on the United Kingdom Parkinson's Disease Society Brain Bank Diagnostic Criteria [9]. At our hospital, gait analysis using a wearable device is recommended for all PD patients at the time of diagnosis, and this analysis is performed only in consenting patients.

The study participants were 53 drug-naive PD patients (29 men and 24 women; median age, 69.0 years). The median disease duration was 0.8 (interquartile range, 0.5–1.3) years (Table 1). The median follow-up period of the study patients was 3.0 years (range, 1.4–5.3). All patients underwent brain magnetic resonance imaging (MRI) or computed tomography (CT) (MRI and CT were performed in 39 and 14 patients, respectively). Since the patients were enrolled in the study with early PD at a time when they had not yet been treated for PD, their diagnosis was confirmed at follow-up in all patients. All patients met the Movement Disorder Society Clinical Diagnostic Criteria for Parkinson's disease [10].

In this study, we classified patients with PD into freezer and nonfreezer groups, based on the clinical assessment at the time of PD diagnosis (Table 1). Clinical evaluation, including FOG assessment and gait analyses, was performed at the time of PD diagnosis.

The study protocol was reviewed and approved by the Medical Ethics Committee of Tokyo Medical University Hospital (#T2020-0110). Signed informed consent was obtained from each participant.

### 2.2. Definition of Freezing and Assessment of FOG Severity

Patients were identified as freezers if they responded positively to item 3 of the Freezing of Gait Questionnaire (FOG-Q3): “Do you feel like your feet are glued to the floor while walking, making a turn, or when trying to initiate walking (freezing)?” [11]. The FOG-Q is a validated screening instrument for the identification of freezers [11, 12]. The severity of FOG was assessed by the total score of items 3–6 of the 6-part FOG-Q, which are directly related to FOG. These include the following: Q3: Do you feel like your feet are glued to the floor while walking, making a turn, or when trying to initiate walking (freezing)? Q4: How long is the longest freezing episode? Q5: How long is the typical start hesitation episode (freezing when initiating the first step)? and Q6: How long is the typical turning hesitation (freezing when turning)? For answers to these questions, a higher total score denoted more severe FOG.

### 2.3. Clinical Assessment

Global cognition was assessed using the MMSE [13]. The Trail Making Test Part A (TMT-A) was used to assess visual scanning, numeric sequencing, and visual-motor speed and part B (TMT-B) was used to evaluate general frontal lobe function [14, 15]. The difference between the time required for completing the TMT-A and TMT-B (TMTB-A) was used to evaluate shifting abilities [14, 15]. The Clock Drawing Test (CDT) was used to evaluate the visuospatial function, according to the method developed by Rouleau et al. (0 = worst performance, 10 = best performance) [16]. The Frontal Assessment Battery (FAB) [17] and the Behavioral Assessment of the Dysexecutive Syndrome (BADS) [18] were used to assess the frontal/executive function. The BADS comprises six subtests, each of which yields a profile score of 0–4 (0 = worst performance, 4 = best performance); the total profile score is the sum of the six subtest scores. The total profile score was converted to a standardized score with a mean of 100 and a standard deviation of 15, based on data obtained from control subjects. The results were expressed as age-adjusted standardized scores, which were calculated by converting the total profile score to a standard score followed by adjustment for age [19].

With regard to psychiatric symptoms, depressive symptoms and apathy were evaluated using the Japanese versions of the Beck Depression Inventory, second edition (BDI-II) [20, 21], and the Starkstein Apathy Scale (AS) [22, 23], respectively. The olfactory function was assessed using the Odor Stick Identification Test for Japanese (OSIT-J) (Daiichi Yakuhin Sangyo Co. Tokyo, Japan), during which the participant must identify 12 different odorants familiar to the Japanese population [24, 25]. The total number of correct answers for the 12 odorants constitutes the OSIT-J score [24, 25]. Motor severity was assessed using the modified Hoehn & Yahr (HY) stage and the Unified Parkinson's Disease Rating Scale (UPDRS) Part III scores [26]. In addition, in the UPDRS assessment, postural instability and gait disorder (PIGD) scores (the sum of scores on UPDRS “PIGD items” 13–15, 29, and 30) and the tremor score (the sum of scores on UPDRS “tremor items” 16, 20, 21) were assessed separately [27].

### 2.4. Gait Analysis Using a Wearable Device and Assessment of Physical Activity

#### 2.4.1. Equipment and Measurements

The MIMAMORI-Gait (LSI Medience Corporation, Tokyo) is a wearable device (size: 8 × 6 × 2 cm; weight: 80 g) that measures, in three dimensions (*a*_x_, *a*_y_, and *a*_z_), the acceleration induced by limb and trunk movements and step-in and kick-off during walking [28–31]. The device was secured with a belt to the front and center of the subject's waist. While standing in the anatomical position, the orientation of the *X*, *Y*, and *Z* axes was medial/lateral, vertical, and anterior/posterior, respectively. Positive *X* values corresponded to the leftward acceleration, positive *Y* values corresponded to the upward acceleration, and positive *Z* values corresponded to the forward acceleration. The device recorded the measurements at a sampling rate of 10 ms and the sensor resolution is approximately 0.16 m/s^2^. On completion of the recording, the absolute values of the acceleration vectors (*a*; *a*^2^ = *a*_x_^2^ + *a*_y_^2^ + *a*_z_^2^) were computed offline and displayed graphically on the PC monitor [28–31].

The recording was performed for a continuous 24 h period commencing between 10 : 00 am and 12 : 00 pm. The participant was instructed to wear the device always over a 24 h period, except when changing clothes or taking a shower/bath.

#### 2.4.2. Calculation of the Amount of Daily Movement

After the continuous 24 h recording, the accelerations induced by all movements were averaged every 10 min, and the data were displayed graphically. Assuming that the curve would fit a gamma distribution, the mean value of the distribution was calculated mathematically. The gamma distribution is defined by the following formula:

(1) $$\begin{array}{c} f\left(x\right)=\frac{\left(x^{k-1}e^{-\left(x/\theta \right)}\right)}{\left(\Gamma \left(k\right)\theta^{k}\right)}\text{for},\;x>0. \end{array}$$

The mean value represents the amount of movement in 24 h, which is an index of daily physical activity [32, 33].

#### 2.4.3. Gait Cycle Duration and Gait Acceleration Amplitude

The acceleration vectors due to stepping are distinguishable from those caused by other limb and trunk movements or unexpected artifacts, based on the previously reported mathematical method of “pattern matching” [28–31]. First, attention focused on a relatively strong signal region (e.g., *a* >1 m/s^2^) in the acceleration time series, and a three-dimensional template wave (*a*_x_, *a*_y_, *a*_z_) with a duration of about 0.5 s was arbitrarily chosen. The cross-correlation *CC*(*t*) between this wave and another wave with a time shift *t* chosen from the whole time series was then computed using the following formula:

(2) $$\begin{array}{c} CC\left(t\right)=\frac{\left(1/p\right)\sum_{i=1}^{p}\left[a_{x}\left(i\right)a_{x}\left(i+t\right)+a_{y}\left(i\right)a_{y}\left(i+t\right)+a_{z}\left(i\right)a_{z}\left(i+t\right)\right]}{{\left\{\left(1/p\right)\sum_{i=1}^{p}\left[a_{x}{\left(i\right)}^{2}+a_{y}{\left(i\right)}^{2}+a_{z}{\left(i\right)}^{2}\right]\right\}}^{\left(1/2\right)}{\left\{\left(1/p\right)\sum_{i=1}^{p}\left[a_{x}{\left(i+t\right)}^{2}+a_{y}{\left(i+t\right)}^{2}+a_{z}{\left(i+t\right)}^{2}\right]\right\}}^{\left(1/2\right)}}. \end{array}$$

Here, *t* is the time index and *p* is the length of the template wave. If the change in acceleration is caused by gait motion, the *CC*(*t*) peaks exhibit alternate changes in magnitude over time due to the left/right body sway during walking [28–31].

The gait cycle duration and acceleration amplitude were measured from the gait-induced acceleration signals. The gait cycle was defined as the time between successive contacts of the same foot with the ground. Since gait accelerations correlate with ground reaction forces, we used the gait acceleration amplitude as an index of ground reaction forces. The duration of the gait cycle and the amplitude of gait-related accelerations were averaged for each 10-min recording. The mean gait cycle duration and gait acceleration for each participant were calculated for the entire 24 h period [28–31].

### 2.5. Statistical Analysis

The clinical parameters were subjected to distribution testing using the Shapiro–Wilk test, and normally distributed parameters were expressed as the mean ± standard deviation (SD), whereas, parameters with skewed distribution were expressed as the median (interquartile range, IQR). Pearson's Chi-squared test, Student's *t*-test, and the Mann–Whitney *U* test were used to compare the clinical parameters of freezers and nonfreezers. For the freezers cohort, we analyzed the relationships between FOG severity (FOG-Q3-6 score) and patient demographics, neuropsychological parameters, and severity of motor symptoms using Spearman's rank correlation coefficient. *P* values < 0.05 denoted statistical significance. All statistical analyses were performed by using IBM SPSS Statistics (version 22.0, IBM Corp, Armonk, NY).

## 3. Results

### 3.1. Comparison between Freezers and Nonfreezers

FOG was observed in eight of 53 patients with PD (4 men and 4 women; mean age, 68.1 ± 7.8 years) (Table 1). Differences in age, sex, side of onset, and disease duration were not significant between freezers and nonfreezers. The neuroradiological findings were as follows: one patient (freezer) exhibited asymptomatic lacunar infarction in the anterior limb of the internal capsule on CT and two (non-freezers) exhibited asymptomatic lacunar infarction in the subcortical white matter in the occipital lobe and the putamen, respectively, on MRI. The MMSE, TMT-A, TMT-B, TMTB-A, CDT, FAB, BADS, BDI-II, and OSIT-J scores also did not differ significantly. On the other hand, the AS score was significantly higher in freezers than in nonfreezers (*p*=0.018). With regard to motor symptoms, the modified HY stage (*p* < 0.001), UPDRS part III score (*p* < 0.001), and PIGD score (*p* < 0.001) were significantly higher in freezers than in nonfreezers. No significant difference in the tremor score was observed between the two groups. Gait analysis showed no significant difference in the mean gait cycle duration, while the mean gait acceleration amplitude was significantly lower in freezers than in nonfreezers (*p*=0.006). Moreover, the amount of daily movement in freezers was significantly lower than that in nonfreezers (*p* < 0.001) (Table 1).

### 3.2. Relationship between FOG Severity and Clinical Parameters in Freezers

The FOG-Q3–6 total score was not significantly correlated with any among the following: MMSE (*p*=0.183), TMT-A (*p*=0.801), TMT-B (*p*=0.491), TMTB-A (*p*=0.801), CDT (*p*=0.096), FAB (*p*=0.750), BADS (*p*=0.641), AS (*p*=0.683), and BDI-II (*p*=0.200) scores. Regarding motor symptoms, there was no significant correlation between the FOG-Q3-6 score and any of the following: modified HY stage (*p*=0.580), UPDRS part III score (*p*=0.275), tremor score (*p*=0.309), and PIGD score (*p*=0.988) (Table 2).

## 4. Discussion

Generally, FOG is considered to occur in patients with advanced PD, with the average onset duration from PD to FOG being 8.1 ± 6.3 years [1, 2]. In the DATATOP cohort study conducted in patients with mild untreated PD, FOG was observed in 7.1% of patients, as determined by the FOG-specific questions of the UPDRS ADL section [34]. In a longitudinal study of patients with early untreated PD without FOG, self-reported FOG was recorded in 16.13%, 39.52%, and 51.61% of patients at 1, 2, and 3 years of treatment, respectively [35]. In the present study, patients with untreated PD within 2 years of disease onset were included, and self-reported FOG was found in 15% of these patients. These results indicate that FOG can occur soon after disease onset and this possibility should be carefully considered in the management of patients with early-stage PD.

In this study, freezers had a significantly higher AS score than nonfreezers. Mood disorders, depression, apathy, and anxiety have been shown to be associated with FOG [36–39]. The results of this study suggest the involvement of apathy in FOG development at an early stage after PD onset. Conversely, global cognition, executive function, and olfactory function did not significantly differ between freezers and nonfreezers. Previous studies have suggested the involvement of cognitive impairment, especially executive dysfunction, in the development of FOG in PD [1, 37]. Moreover, a longitudinal study of patients with early-stage PD showed that impaired processing speed and verbal learning are predictive factors for FOG [40]. Further investigations are needed to clarify the relationship between FOG and cognitive dysfunction in early-stage PD.

In this study, the severity of motor symptoms, especially the PIGD score, was significantly higher and the mean gait acceleration amplitude was significantly lower in freezers compared to nonfreezers. In the DATATOP cohort study, patients with FOG had significantly higher UPDRS motor scores except for tremors, and speech and gait impairment was associated with the presence of FOG [34]. A recent study reported that 9–16% of patients with PD have a treatment-refractory, rapid-progressing disease subtype (diffuse malignant type) characterized by severe motor symptoms, early gait problems, and nonmotor symptoms, such as rapid eye movement sleep disorders and orthostatic hypotension [41]. Although it is unclear whether the subgroup of patients with PD presenting with FOG at an early stage are included in this subtype population, future studies should include longitudinal evaluation of other nonmotor symptoms and treatment responses to elucidate the pathogenesis of FOG.

Patients with PD have reduced daily physical activity soon after disease onset [33]. Our study showed that FOG at least in part plays a role in the reduced daily physical activity in patients with early-stage PD, suggesting the importance of appropriate evaluation and management of FOG in patients with early-stage PD.

In this study, analysis of the association of FOG severity with various neuropsychological parameters and severity of motor symptoms in freezers showed no significant correlations between FOG severity and these parameters, including the AS score, modified HY stage, UPDRS Part III score, and PIGD score. This finding suggests that the assessment of the FOG-Q 3–6 total score does not sufficiently reflect FOG severity because of the small number of freezers and relatively mild FOG in freezers. In addition, the duration of FOG appeared to be an important factor in early-stage PD. A multifaceted assessment of severity, incorporating these factors, appears necessary.

Various pathological conditions are involved in the development of FOG [1]. Disorders of nondopaminergic systems, such as the acetylcholine system, have been reported to be involved in the development of FOG [42]. Thus, FOG, particularly when it appears with the progression of the disease stage, is considered to have a heterogeneous pathology [1]. In fact, the response to antiparkinsonian drugs, such as levodopa, also varies among patients [37, 43]. In contrast, early-onset FOG appears to arise from relatively homogeneous pathology. Therefore, investigation of such pathology might be important for elucidating the pathogenic mechanism of FOG. The present study, which included patients with untreated early-stage PD, demonstrated that the development of early-onset FOG is associated with gait disturbance and impaired postural control, similar to previous studies [34]. The Parkinson's Progression Markers Initiative study, which was conducted in patients with early-stage PD, reported that presynaptic striatal dopaminergic depletion shown by dopamine transporter imaging can predict the development of FOG [44]. These results suggest that the essential pathology of early-onset FOG is motor symptoms caused by striatal dopaminergic denervation, particularly gait disturbance and impaired postural control and that FOG occurrence, may depend on the severity of these symptoms. In this study, the AS score was significantly higher in freezers. Interestingly, studies that included patients with untreated early-stage PD have reported that apathy is associated with striatal dopaminergic innervation and also significantly associated with PIGD scores [45, 46]. Furthermore, studies are necessary to investigate how apathy is involved in the development of FOG.

Our study has several limitations. First, only 53 patients with untreated early-stage PD were included, which was a smaller sample size compared to that in previous studies. This is because the study only included patients with PD within 2 years of disease onset and had strict exclusion criteria to eliminate factors other than PD, especially those related to complications, as much as possible. In addition, patients aged under 80 years were included in the present study considering the physical burden of wearing the device for 24 hours. The second limitation is related to FOG evaluation. In this study, the presence or absence of self-reported FOG was evaluated using the FOG-Q3, and its severity was evaluated using the FOG-Q3–6 scores. Although similar approaches were also adopted in earlier studies, evaluations using the FOG-Q are based on patients' subjective judgment and are not clinically validated. Third, cognitive function was evaluated mainly by global cognition and executive dysfunction measures. In PD, other cognitive domains, such as attention, working memory, language, memory, and visuospatial function, are also known to be impaired at an early disease stage [47]. Future studies should evaluate other cognitive domains and determine their relationship with FOG. Lastly, we cross-sectionally examined the FOG characteristics in untreated early-stage PD patients in this study. In the future, longitudinal studies, such as treatment response studies, are warranted.

## 5. Conclusions

Self-reported FOG was observed in 15% of newly diagnosed and levodopa-naïve patients with PD. Freezers had a significantly higher AS score and greater severity of motor symptoms, especially gait disturbances, compared with nonfreezers. FOG was an independent factor associated with reduced daily physical activity in patients with PD.

## Figures and Tables

**Table 1:** Comparison of clinical parameters between FOG and non-FOG patients.

	Total PD (*n* = 53)	Freezers (*n* = 8)	Nonfreezers (*n* = 45)	*pp* value
Demographic				
Age (years)	69.0 (64.0−72.0)	68.1 ± 7.8	69.0 (64.0−71.0)	0.471
Sex (male/female)	29/24	4/4	25/20	0.534^*∗*^
Side of onset (right/left)	29/24	4/4	25/20	0.534^*∗*^
Disease duration (years)	0.8 (0.5−1.3)	0.8 ± 0.4	0.8 (0.5−1.3)	0.951
MRI/CT abnormal findings (n)	3	1	2	0.364
Assessment scale				
MMSE score	28.0 (27.0−29.0)	27.0 (27.0−29.0)	29.0 (26.0−29.0)	0.583
TMT-A	45.5 (36.8−57.1)	57.5 ± 14.8	44.4 (36.2−50.8)	0.056
TMT-B	114.5 (91.8−157.2)	133.9 ± 28.4	114.0 (91.0−156.7)	0.399
TMTB-A	67.1 (52.1−96.4)	76.4 ± 21.7	66.5 (52.0−96.3)	0.655
CDT	10.0 (10.0−10.0)	10.0 (10.0−10.0)	10.0 (10.0−10.0)	0.989
FAB	15.0 (14.0−17.0)	15.0 (13.0−15.0)	16.0 (14.0−17.0)	0.086
BADS	103.9 ± 14.4	94.1 ± 14.1	105.4 ± 14.0	0.054
BDI-II	5.0 (4.0−10.0)	9.0 ± 4.3	5.0 (2.0−7.0)	0.063
AS	9.0 (5.0−16.0)	15.5 ± 4.1	8.0 (4.0–13.0)	0.018
OSIT-J	5.3 ± 2.7	5.6 ± 1.9	5.3 ± 2.8	0.785
Modified HY stage	2.0 (2.0−2.5)	3.0 (2.8−3.0)	2.0 (1.5−2.0)	<0.001
UPDRS part III score	13.0 (9.0−18.0)	30.0 ± 10.9	11.0 (7.0−16.0)	<0.001
Tremor score	2.0 (1.0−4.0)	1.5 (1.0−2.0)	3.0 (1.0−4.0)	0.321
PIGD score	2.0 (0.0−3.0)	6.6 ± 1.8	1.0 (0.0−2.0)	<0.001
FOG-Q	2.0 (1.0−3.0)	10.1 ± 2.5	2.0 (0.0−3.0)	<0.001
Gait analysis				
Mean gait cycle duration	1.22 ± 0.10	1.18 ± 0.12	1.23 ± 0.10	0.245
Mean gait acceleration amplitude	1.79 ± 0.39	1.45 ± 0.29	1.85 ± 0.38	0.006
Amount of daily movement	0.41 ± 0.12	0.28 ± 0.09	0.43 ± 0.11	<0.001

Data are median (interquartile range) or a number of patients. *P* values by the Mann–Whitney *U* test, except for those marked with an asterisk (^*∗*^), which are by Pearson's chi-squared test. FOG: Freezing Of Gait; MMSE: Mini-Mental State Examination; TMT-A: Trail Making Test-A; TMT-B:Trail Making Test-A; TMT-B-A:Trail Making Test Part B minus Part A; CDT: Clock Drawing Test; FAB: Frontal Assessment Battery; BADS: Behavioral Assessment of the Dysexecutive Syndrome; BDI-II: Beck Depression Inventory, Second Edition; SAS: Starkstein Apathy Scale; OSIT-J: Odor Stick Identification Test for Japanese; Modified HY stage: modified Hoehn and Yahr stage; UPDRS part III score: Unified Parkinson's Disease Rating Scale part III score; Tremor score: the sum of UPDRS “tremor items” 16, 20, and 21; Postural instability and gait disorder (PIGD) score: the sum of UPDRS “PIGD items” 13–15, 29, and 30; FOG-Q: Freezing of Gait Questionnaire.

**Table 2:** Association of the FOG-Q 3–6 total scores with neuropsychological test performance, olfactory function, and severity of motor symptoms.

Measure	FOG-Q 3–6 total score	
	*ρ*	*p* value
Age	−0.025	0.952
Duration	0.332	0.422
MMSE	0.524	0.183
TMT-A	−0.118	0.801
TMT-B	−0.315	0.491
TMTB-A	−0.118	0.801
CDT	−0.676	0.096
FAB	0.149	0.750
BADS	−0.217	0.641
BDI-II	0.507	0.200
AS	0.172	0.683
OSIT-J	−0.123	0.793
Modified HY stage	0.232	0.580
UPDRS part III score	0.440	0.275
Tremor score	−0.413	0.309
PIGD score	0.006	0.988

Data are Spearman's rank correlation coefficient (*ρ*). Abbreviations are as shown in Table 1.

## Data Availability

The data that support the findings of this study are not publicly available because they contain information that could compromise the privacy of the research participants but are available from the corresponding author (HT) upon reasonable request.
